# Supplementary material for: Geographical variation and factors associated with unsafe child stool disposal in Ethiopia: A spatial and multilevel analysis
Source: PLoS One. 2021 Apr 29;16(4):e0250814. doi: 10.1371/journal.pone.0250814 (PMC8084221; doi:10.1371/journal.pone.0250814)
Supplement: S1 File — (DOCX) [file pone.0250814.s001.docx]

**S1 File. Basis for categorizing independent variables**

| **Individual-level factors (Level 1)** | **Category** | **Type of variable** | **Description** |
| --- | --- | --- | --- |
| **Child characteristics** |  |  |  |
| Sex of the child | Categorized in to (1) male; or (2) female | Binary | Sex of the child was a binary variable categorized as male or female |
| Age of child (months) | Categorized into (1) 0–5; (2) 6–11; (3) 12–17; and (4) 18–23 | Categorical variable | The EDHS collected data on age of the child in months. In the current study, age of the child was coded into four age categories as 0-5 months; 6-11 months; 12-17 months; and 18-23 months. Age categorization was guided by the previous workers of literature. |
| Diarrhea in the last two weeks | Categorized into (1) yes; or (2) no | Binary | The EDHS collected data on the occurrence of diarrhea in children below five years of age, mothers were asked ‘Has (NAME) had diarrhea in the last two weeks?’ The response was recoded as yes or no. |
| **Maternal/paternal/household**  **Characteristics** |  |  |  |
| Maternal age in years | Categorized into (1) 15-24; (2) 25-34; and ≥ 35 | Categorical variable | The EDHS collected data on maternal age in years. In the current study, maternal age was coded into three age category as 15-24; 25-34; ≥ 35 following the previous workers of literature. |
| Educational level of mother | Categorized in to (1) no formal education; (2) primary; (3); secondary; or (4) higher | Categorical variable | The EDHS collected data on women educational level as no formal education, primary; secondary and higher. |
| Mother’s employment status | Categorized in to (1) not employed, or (2) employed | Binary | The EDHS collected data on women employment as ‘no job’ or as a list of different jobs. The sorts of jobs collected for women ranged from unskilled manual to professional. In the current study, the responses were coded into ‘not employed=no job’ or ‘employed/have job’ regardless of the type of job. The ‘not working/no job’ category does not mean that women do not perform any activities; rather, women are limited to household activities. |
| Number of under-five children | Categorized into (1) ≤ 2; or (2) ≥ 3 | Binary | The EDHS collected data on number of under-five children. In the current study, number of under-five children was categorized as (1) ≤ 2; or (2) ≥ 3 following the previous workers of literature. |
| Wealth index | Categorized into (1) (first quintile) (poorest); (2) (second quintile) (poorer); (3) (third quintile) (middle); (4) (fourth quintile) (richer);or (5) (fifth quintile) (richest) | Categorical variable | Households are given scores based on the number and kinds of consumer goods they own, ranging from a television to a bicycle or car, in addition to housing characteristics such as source of drinking water, toilet facilities, and flooring materials. These scores are derived using principal component analysis. Household wealth index categorized in quintiles: poorest, poorer, middle, richer and richest. The index was constructed using household asset data by using a principal components analysis (PCA). |
| Source of drinking water | Categorized in to (1) improved; or (2) unimproved | Binary | This variable in the EDHS can categorized as improved and unimproved water sources. Improved sources of drinking water include piped water, public taps, standpipes, tube wells, boreholes, protected dug wells and springs, and rainwater. Because the quality of bottled water is unknown, households that use bottled water for drinking are classified as using an improved source only if the water they use for cooking and hand washing comes from an improved source. |
| Latrine type | Categorized in to (1) improved; or (2) unimproved | Binary | This variable in the EDHS can broadly categorized as improved and unimproved toilet facility. Improved toilet facilities include any non-shared toilet of the following types: flush/pour flush toilets to  piped sewer systems, septic tanks, and pit latrines; ventilated improved pit (VIP) latrines; pit latrines with slabs; and composting toilets. Any facility other than the aforementioned toilet facilities were considered as unimproved. |
| Community-level factors (Level 2) |  |  |  |
| Place of residence | Categorized into (1) urban; or (2) rural | Binary | This variable in the EDHS can explain characteristics of the clusters directly. The two categories were: urban; rural. |
| Region | Categorized in to (1) agrarian; (2) pastoralist or (3) city | Categorical variable | Contextual region: The interest of the current study was not in the regions delineated for administrative purpose, which might not necessarily be related to child stool disposal of the population. In the current study the regions were categorized into agrarian, pastoralist, and city, based on their settings that may have a relationship with health-seeking behavior, accessibility to health service, and sanitation facility. The regions of Tigray, Amhara, Oromiya, SNNP, Gambella, and Benshangul Gumuz were recorded as agrarian. The Somali and Afar regions were combined to form the pastoralist region and the city administrations- Addis Ababa, Dire Dawa, and Harar were combined as city. Though Gambela and Benshangul Gumuz have been considered as pastoralists since recent times, their living settings actually approached the agrarian. |
| Community poverty | Categorized in to (1) high; or (2) low | Binary | Community poverty level was generated by aggregating the individual characteristics in a cluster since EDHS did not collect data that can directly describe the characteristics of the clusters except the place of residence. The aggregate community poverty level was constructed by aggregating individual level characteristics at the community (cluster) level. Accordingly, community poverty level was an aggregate wealth index categorized as high or low, which is the proportion of women in the poorest and poorer quintile derived from data on wealth index which is categorized as low and high poverty community. Since the aggregate community poverty level value is not normally distributed, it was categorized into high and low groups based on the median value. Community poverty level was categorized as high if the proportion of women from the two lowest wealth quintiles in a given community was higher than the median value and low if the proportion was less than and equal to median value. |
