# Supplementary material for: Geographical variation and factors associated with unsafe child stool disposal in Ethiopia: A spatial and multilevel analysis
Source: PLoS One. 2021 Apr 29;16(4):e0250814. doi: 10.1371/journal.pone.0250814 (PMC8084221; doi:10.1371/journal.pone.0250814)
Supplement: S2 File — (DOCX) [file pone.0250814.s002.docx]

**S2 File. Significant spatial clusters of unsafe child stool disposal in Ethiopia, enumeration areas(clusters) detected and there Coordinates/radius.**

| **Clusters (n)** | **Enumeration areas(clusters) detected** | **Coordinates/radius** | **Population** | **Cases** | **RR** | **LLR** | **P-value** |
| --- | --- | --- | --- | --- | --- | --- | --- |
| 1 (201) | 425, 80, 551, 188, 340, 628, 579, 156, 322, 575, 636, 258, 181, 584, 152, 538, 597, 400, 542, 312, 98, 590, 81, 355, 424, 583, 255, 327, 528, 199, 84, 430, 481, 638, 604, 612, 392, 640, 45, 160, 237, 94, 550, 78, 461, 605, 66, 136, 268, 220, 143, 384, 296, 300, 129, 226, 479, 623, 89, 449, 341, 99, 298, 504, 79, 598, 163, 421, 253, 404, 196, 128, 442, 97, 511, 351, 127, 413, 117, 192, 130, 512, 401, 132, 362, 235, 172, 263, 279, 585, 488, 200, 103, 455, 292, 158, 478, 627, 591, 249, 134, 169, 456, 344, 332, 73, 545, 38, 544, 431, 241, 599, 496, 167, 189, 389, 516, 403, 382, 354, 571, 24, 348, 120, 191, 52, 616, 429, 611, 176, 345, 361, 259, 18, 617, 254, 206, 460, 602, 368, 415, 109, 541, 10, 3, 427, 482, 386, 375, 548, 55, 267, 515, 474, 615, 498, 205, 570, 547, 531, 499, 178, 334, 350, 510, 276, 533, 310, 246, 218, 620, 559, 572, 494, 637, 36, 440, 283, 632, 150, 596, 423, 183, 102, 295, 184, 256, 137, 37, 4, 35, 75, 244, 135, 366, 201, 336, 484, 517, 320, 399 | (13.351814 N, 38.353591 E) / 471.07 km | 1,272 | 931 | 1.26 | 41.62 | < 0.0001 |
| 2^a^ (26) | 618, 266, 309, 435, 536, 370, 507, 592, 260, 104, 233, 69, 426, 603, 346, 315, 13, 567, 343, 105, 417, 284, 106, 265, 593, 270 | (8.238420 N, 33.229506 E) / 147.69 km | 164 | 144 | 1.41 | 26.07 | < 0.0001 |
| 3^b^ (26) | 208, 520, 556, 394, 7, 377, 480, 82, 187, 278, 318, 164, 358, 85, 601, 138, 422, 289, 472, 34, 452, 398, 492, 316, 21, 146 | (4.006703 N, 41.599741 E) / 419.89 km | 256 | 206 | 1.30 | 19.10 | <0.0001 |
| 4^c^ (6) | 277, 568, 527, 22, 116, 33 | (9.107168 N, 43.165843 E) / 45.70 km | 41 | 40 | 1.55 | 14.71 | <0.0001 |
| 5^d^ (3) | 186, 8, 210 | (9.292185 N, 42.553365 E) / 18.63 km | 27 | 27 | 1.59 | 12.41 | <0.001 |
| 6^e^  (8) | 476, 506, 412, 122, 333, 245, 372, 529 | (8.888553 N, 40.744565 E) / 63.62 km | 81 | 70 | 1.38 | 11.07 | <0.001 |

^a^ The secondary clusters’ were typically located in the Gambela region and centered at 8.238420 N, 33.229506 E with 147.69 km radius, RR: 1.41, and LLR of 26.07 at p-value < 0.0001. It shows that children within the area had 1.41 times higher risk of unsafe child stool disposal than outside the area. ^b^The third clusters’ were located in Oromia (south) and Somali (southeast) regions and centered at 4.006703 N, 41.599741 E) with 419.89 km radius, RR: 1.30 and LLR of 19.10 at p-value < 0.0001. ^c^The fourth clusters’ were located in Somali (north) regions and centered at 9.107168 N, 43.165843 E with 45.70 km radius, RR: 1.55, and LLR of 14.41 at p-value < 0.0001. ^d^The fifth clusters’ were typically located in Hareri regions and centered at 9.292185 N, 42.553365 E with 18.63 km radius, RR: 1.59, and LLR of 14.41 at p-value < 0.001. ^e^The six clusters’ were typically located in Oromia (northeast) regions and centered at 8.888553 N, 40.744565 E with 63.62 km radius, RR: 1.38, and LLR of 11.07 at p-value < 0.001.
