# Supplementary material for: Geographical variation and factors associated with unsafe child stool disposal in Ethiopia: A spatial and multilevel analysis
Source: PLoS One. 2021 Apr 29;16(4):e0250814. doi: 10.1371/journal.pone.0250814 (PMC8084221; doi:10.1371/journal.pone.0250814)
Supplement: S1 Fig — (PDF) [file pone.0250814.s003.pdf]

Spatial Autocorrelation Report

Moran's Index: 0.210617

z-score: 84.417876

p-value: 0.000000

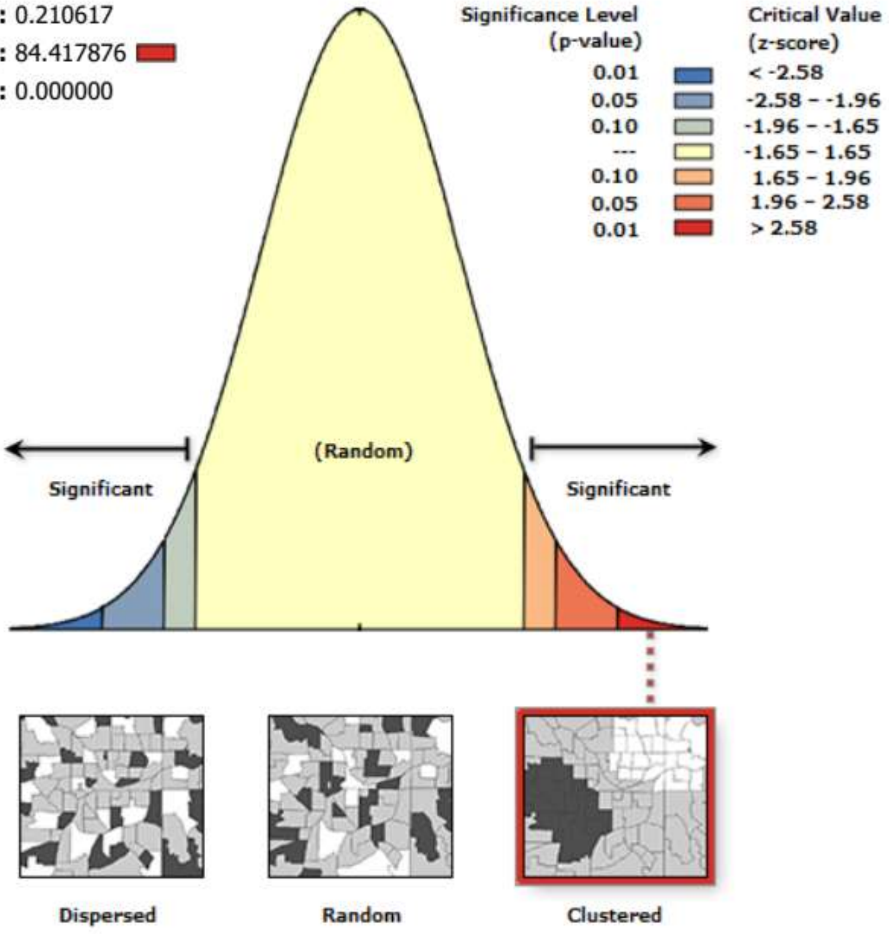

Given the z-score of 84.4178761958, there is a less than 1% likelihood that this clustered pattern could be the result of random chance.
