# Supplementary material for: Geographical variation and factors associated with unsafe child stool disposal in Ethiopia: A spatial and multilevel analysis
Source: PLoS One. 2021 Apr 29;16(4):e0250814. doi: 10.1371/journal.pone.0250814 (PMC8084221; doi:10.1371/journal.pone.0250814)
Supplement: Attachment — Submitted filename: PONE-D-20-24200_review 2020.docx [file pone.0250814.s004.docx]

Geographical variation and factors associated with unsafe child stool disposal in Ethiopia: A spatial and multilevel analysis

Very well written manuscript. This is really a very important and timely work as in the LMICs still struggling with open defection and child feces disposal. Very few works done in this area and I really appreciate authors for this work.

I really like the way presented the findings as well as noticed the limitation of the study. However, few minor comments might improve the manuscript.

1. Author didn’t mention the name of the institution where received Institutional Review Board approval.
2. Vast majority of the mother 60.3% had no formal education, so it would be great to include whether any association of unsafe child feces disposal practices with mother’s education.
3. Having the toilet facilities is important to promote safe disposal, however if any household don’t have latrine how you considered in this analysis?
