# Supplementary material for: Geographical variation and factors associated with unsafe child stool disposal in Ethiopia: A spatial and multilevel analysis
Source: PLoS One. 2021 Apr 29;16(4):e0250814. doi: 10.1371/journal.pone.0250814 (PMC8084221; doi:10.1371/journal.pone.0250814)
Supplement: Attachment — Submitted filename: Review 001.docx [file pone.0250814.s005.docx]

**Manuscript Title:**

Geographical variation and factors associated with unsafe child stool disposal in

Ethiopia: A spatial and multilevel analysis

**Reviewer’s decision**

The manuscript is of high quality considering the amount of work-done and analysis made. It is also well written. However, some minor corrections needs to be made prior and returned for another round of review before consideration for publication.

**Summary of Reviewer’s comment:**

This is a very important and interesting research. The introduction is sound and well structured. The rationale and objectives of the study are clearly spelt out. The methodology section is well written but needs improvement. Authors need to consider the introduction of new subsections, and provide more clarity on the categorization of variables. The data management and analysis plan was described appropriately. The data analysis section is robust, and the authors have done a very great job at pulling all the data together. However, there are issues with results presentation and description within text for a particular table. In addition, concerns on why the authors decided to pull the administrative regions together under agarian, pastoralist and city categories before the multivariate logistic analysis needs to be rechecked. The discussion is sound and well written. More specific comments have been raised in the additional file attached and authors should consider working on them.

**Reviewer’s Comment**

**TOPIC:**

The title of this manuscript is appropriate and concise,

**INTRODUCTION**

Line 48: recast as..”….open fields, garbages, drainages and burying in soils are considered unsafe as it exposes vulnerable children who interact with such to many fecal-oral diseases.

Line 50: child stool disposal is..

Line 54: Recast as “Futhermore, there are established evidences on the effect …….

Line 58: Replace “with available” with “according to”

Line 58: It is not uncommon that major contributors….

Line 61-62: Move this to Line 58 after the reference [2]

Line 63: previous studies has identified…

Line 69-70: Are the authors trying to discuss the findings of the research ahead of time. If yes, please expunge.

***Materials and Methods***

Authors should restructure their methods section. It is expected that, the authors would provide a as a starting line, a brief summary of the study location in this case, Ethiopia i.e the districts, populations, health system, language, etc.

Then an additional sub-section on study design should be developed. This is where the authors would explain the type of design employed, as it appears a secondary analysis was done.

Authors can then follow up with another section of data source and extraction, where they will provide very explicit details on how data were sourced, and explain in great details the EDHS

The sections on study variables is fine.

Line 114: rephrase as “was guided by already existing literatures.

Line 119: replace recorded with “categorized”

Line 120: replace combined to form the with “categorized as”

Line 121: Replace combined as the with “categorized as”

Line 122-123 (Table 1): Can authors please provide descriptive text on what metrics they used in estimating community poverty as low or high. It appears they used wealth index that has been described on this same table. How did they go about the grouping?

Table 1: Authors should provide more descriptive text on how the wealth index was estimated from the source survey. They should provide very clear notes that would guide the readers. Same thing applied to source of drinking water. How did they arrive at the category of improved and unimproved?.

Table 1: Can authors expunge the “categorized into that reflects on all the cell in the category column. I t would be more appropriate to just have the categories listed e.g (1) male (2) female.

Line 125: should start with “Secondary data obtained were imported(?) and analyzed in…….

Line 127-128: …weighting procedure has been sufficiently described in EDHS methodology [10]

Line 129-131: Please recast as follow”………….children within a cluster are more similar………clusters. As such, a multilevel model is……..because it allows analysis based on hierarchical structure of variables”.

Line 145: Remove “ STATA……analyses”.

**RESULTS**

Table 2; Can authors explain and address why some of the demographic variables do no amount to 4145. If there are no response or missing data, they should be described so the readers can have a better understanding

Table 2: Under mothers working status, can authors find a more befitting category rather than the Not working and working used

Table 2: Still very much bothered about the community poverty level here. It appears vague

Table 3: This table has been poorly described. Authors should take their time to explain the details contained in the table. At a first glance, I believe the authors are focusing on the odds of practicing unsafe stool disposal. The results presented here shows varying levels of odds, which I found quite interesting. For instance, the odds of practicing unsafe disposal was reduced as the children increase in age, except for those aged 12-17 months. Looking at the data this way may provide very great insight on how to discuss the results subsequently.

Line 182-197: Since authors knew they would still present and discuss their findings by districts/regions i.e Tigray, Amhara etc, why did they group these administrative regions together in their multivariate logistic model. It would have been great if they analyse them separately too, and have odd ratios that would add credence to the maps they presented as fig 1 and 2.

Table 4: Can the column on enumeration areas be removed. At the moment it adds no value to the table, it would be better if the clusters can be provided as a supplementary file, with aadditional descriptive lines provided. But for this present table, the authors should retain 7 columns. The first can be titled category of clusters i.e primary, secondary etc. while the second column, can be titled number of clusters detected i.e. 201, 26 ..

Line 211-217: The footnotes provided here should rather be converted to main text, and follow with line 207.

Line 224-225: Authors should check the write up. The 39.61% is from the null model not community level factors

Line 225: Please recast as “Furthermore, between -cluster variability….

Table 5: The heading row shoes AOR (95% CI). Please authors should take this out

Table 6: Looks great.

Line 251: insert a parenthesis after middle, before AOR.

**DISCUSSION**

The discussion section is sound, and appropriately written in context of the results presented

**Limitations**

Line 335: Start with Although,

Line 336: expunge “, a model”

Line 341: expunge, in

Line 343: recast as “…..nature of the survey is not appropriate to estimate the cause and effect…….
